# Changes in Inner and Outer Retinal Layer Thicknesses after Vitrectomy for Idiopathic Macular Hole: Implications for Visual Prognosis

**DOI:** 10.1371/journal.pone.0135925

**Published:** 2015-08-20

**Authors:** Yuki Hashimoto, Wataru Saito, Akio Fujiya, Chikako Yoshizawa, Kiriko Hirooka, Shohei Mori, Kousuke Noda, Susumu Ishida

**Affiliations:** 1 Department of Ophthalmology, Hokkaido University Graduate School of Medicine, Sapporo, Japan; 2 Department of Ocular Circulation and Metabolism, Hokkaido University Graduate School of Medicine, Sapporo, Japan; Tohoku University, JAPAN

## Abstract

**Purpose:**

To investigate sequential post-operative thickness changes in inner and outer retinal layers in eyes with an idiopathic macular hole (MH).

**Methods:**

Retrospective case series. Twenty-four eyes of 23 patients who had received pars plana vitrectomy (PPV) for the closure of MH were included in the study. Spectral domain optical coherence tomography C-scan was used to automatically measure the mean thickness of the inner and outer retinal layers pre-operatively and up to 6 months following surgery. The photoreceptor outer segment (PROS) length was measured manually and was used to assess its relationship with best-corrected visual acuity (BCVA).

**Results:**

Compared with the pre-operative thickness, the inner layers significantly thinned during follow-up (*P* = 0.02), particularly in the parafoveal (*P* = 0.01), but not perifoveal, area. The post-operative inner layer thinning ranged from the ganglion cell layer to the inner plexiform layer (*P* = 0.002), whereas the nerve fiber layer was unaltered. Outer layer thickness was significantly greater post-operatively (*P* = 0.002), and especially the PROS lengthened not only in the fovea but also in the parafovea (*P* < 0.001). Six months after surgery, BCVA was significantly correlated exclusively with the elongated foveal PROS (*R* = 0.42, *P* = 0.03), but not with any of the other thickness parameters examined.

**Conclusions:**

Following PPV for MH, retinal inner layers other than the nerve fiber layer thinned, suggestive of subclinical thickening in the inner layers where no cyst was evident pre-operatively. In contrast, retinal outer layer thickness significantly increased, potentially as a result of PROS elongation linking tightly with favorable visual prognosis in MH eyes.

## Introduction

Pars plana vitrectomy (PPV) with internal limiting membrane (ILM) removal has been performed on patients with an idiopathic macular hole (MH), resulting in a high MH closure rate and improvement in visual function [1,2]. Brilliant blue G (BBG) is widely used in the treatment of vitreoretinal diseases because it has been reported to have good ILM staining properties with minimal retinal cytotoxicity [3] in terms of its potential neuroprotective actions [4].

Recent advances in spectral domain optical coherence tomography (SD-OCT) technology, such as the development of B- and C-scans, have made it possible to accurately depict changes in the outer and inner retinal layers of the posterior fundus. The microstructures of the macula have been investigated in association with MH surgery. Recovery of the ellipsoid zone (EZ) and/or the external limiting membrane (ELM) was associated with the best-corrected visual acuity (BCVA) or the foveal sensitivity after surgery [5–8]. Additionally, the length of the pre-operative interdigitation zone (IZ) defect can predict the post-operative BCVA [9]. These observations suggest that the morphology of the outer retinal layers in the fovea is related to the visual function in MH eyes.

There have been some studies examining changes in the inner retinal layer before and after surgery for MH eyes with OCT C-scan. The inner retinal layer thickness from the nerve fiber layer (NFL) to the inner plexiform layer (IPL) was significantly reduced post-operatively in eyes that had received MH surgery, and there was a significant correlation between the inner layer thickness and retinal sensitivity 6 months after surgery [10]. This suggests that there is a relationship between inner retinal thickness and visual function. However, little is known about which layers of the inner retina are most affected as a result of PPV for MH.

In eyes with MH, post-operative changes in retinal thickness appear to involve both the fovea and its surrounding area. Ohta et al. compared changes in the thickness of the retinal layer in both the parafoveal region (between an annulus with diameter of 1 and 3 mm) and the perifoveal region (between an annulus with diameter of 3 and 6 mm) using C-scan in eyes that had received PPV [11]. Post-operatively, the total retinal thickness significantly decreased at the parafovea but significantly increased at the perifovea. However, the specific layers responsible for the thickness changes in these regions have not been examined.

In this study, we present novel data on pre- and post-operative inner and outer layer thickness changes over time following BBG-assisted PPV for MH eyes, together with their correlation with visual prognosis.

## Materials and Methods

### Inclusion Criteria

This retrospective study includes 24 consecutive eyes from 23 patients (13 men, 10 women) who underwent PPV for an idiopathic MH at Hokkaido University Hospital between October 2010 and November 2012 and were followed up with SD-OCT B- and C-scans over a period of at least 6 months. Eyes with an MH size of more than 1,000 μm in diameter or MH associated with other ocular disorders (e.g., trauma, epiretinal membrane [ERM], macular edema, diabetic retinopathy, myopia of severer than –6 diopters, and/or glaucoma) were excluded from the analyses in the present study. As a result, all enrolled eyes showed successful closure of MH post-operatively. Clinical characteristics of the patients are summarized in Table 1. Mean patient age was 62.8 ± 7.5 years (range, 45–78 years) and the post-operative follow-up period averaged 28.7 ± 9.8 months (range, 7–45 months). Ten MH eyes showed Stage 2, while 9 and 5 eyes showed Stages 3 and 4, respectively. This study was approved by the ethics committee of Hokkaido University Hospital (approval ID: 014–0180) and adhered to the tenets of the Declaration of Helsinki. Written informed consent was obtained from all subjects after the nature and possible consequences of the study had been explained.

**Table 1:** Morphological Changes at the Fovea in Patients with MH.

	Age				Follow-up	ELM		EZ		IZ	
Case	(Years)	Sex	Eye	Stage	(Months)	Post 1M	Post 6M	Post 1M	Post 6M	Post 1M	Post 6M
1	70	M	L	2	45	±	+	-	±	-	-
2	56	F	R	3	40	±	+	±	+	-	+
3	63	M	R	2	40	±	+	-	+	±	±
4	66	M	R	2	40	±	+	±	±	-	-
5	62	F	L	4	38	±	±	±	±	-	±
6	56	F	L	3	38	±	±	±	±	-	-
7	75	F	R	3	37	-	-	-	-	-	-
8	63	M	L	2	36	+	+	±	+	-	±
9 (R)	78	M	R	2	36	±	±	±	±	-	-
9 (L)			L	2	36	±	±	±	±	±	±
10	68	M	L	4	35	±	+	±	+	-	-
11	67	M	R	3	28	±	±	±	±	-	±
12	59	M	R	4	29	±	±	±	±	-	±
13	62	F	R	2	26	+	+	±	+	-	+
14	61	M	R	3	25	±	+	±	+	-	±
15	76	M	R	2	21	±	±	±	+	±	±
16	62	F	L	3	23	+	+	-	+	-	±
17	63	F	L	3	21	±	±	±	±	±	±
18	60	M	L	3	20	-	±	-	±	-	-
19	51	F	L	2	19	+	+	-	±	-	-
20	57	F	R	4	18	±	±	-	±	-	-
21	67	M	L	3	17	+	+	-	+	-	±
22	57	M	L	2	15	±	+	-	±	-	±
23	45	F	R	4	7	±	+	±	+	-	+

MH = macular hole; ELM = external limiting membrane; EZ = ellipsoid zone; IZ = interdigitation zone;

(+) normal;

(±) discontinuous;

(-) invisible

### Surgical Procedures

Twenty-four eyes underwent standard three-port transconjunctival PPV using a 23- or 25-gauge trocar system. Four surgeons (W.S., A.F., C.Y., and S.I.) performed PPV for these eyes. The surgical procedures included the intentional generation of posterior vitreous detachment except in eyes with Stage 4 MH, followed by vitreous gel excision to the equator using triamcinolone acetonide. In all cases, the ILM was peeled off in a range of at least more than three disc diameters with vitreous forceps after being stained with BBG. A sulfur hexafluoride (SF_6_) gas tamponade was applied in all patients, who were instructed to remain in a prone position for 3 days following surgery. Immediately after surgery, 2 mg of subconjunctival dexamethasone was given and subjects were instructed to use topical 0.1% betamethasone 3 times a day for 3 months. Cataract surgery was also performed at the time of PPV in 23 phakic eyes. No patients suffered postoperative complications that potentially affect OCT imaging during follow-up, such as hazy cornea, dislocated intraocular lens, or posterior capsule opacity. In one patient, retinal photocoagulation was performed during surgery to treat iatrogenic retinal breaks.

### Ophthalmic Evaluations

All patients underwent thorough ophthalmologic examinations before surgery and 1, 3, and 6 months post-operatively. The examinations included slit-lamp microscopy, ophthalmoscopy, color fundus photography, spectral domain OCT (RS-3000 and RS-3000 Advance with a software version NAVIS-EX 1.3.7; NIDEK, Gamagori, Japan), and BCVA measurements with a Japanese standard Landolt visual acuity chart.

**Fig 1:**
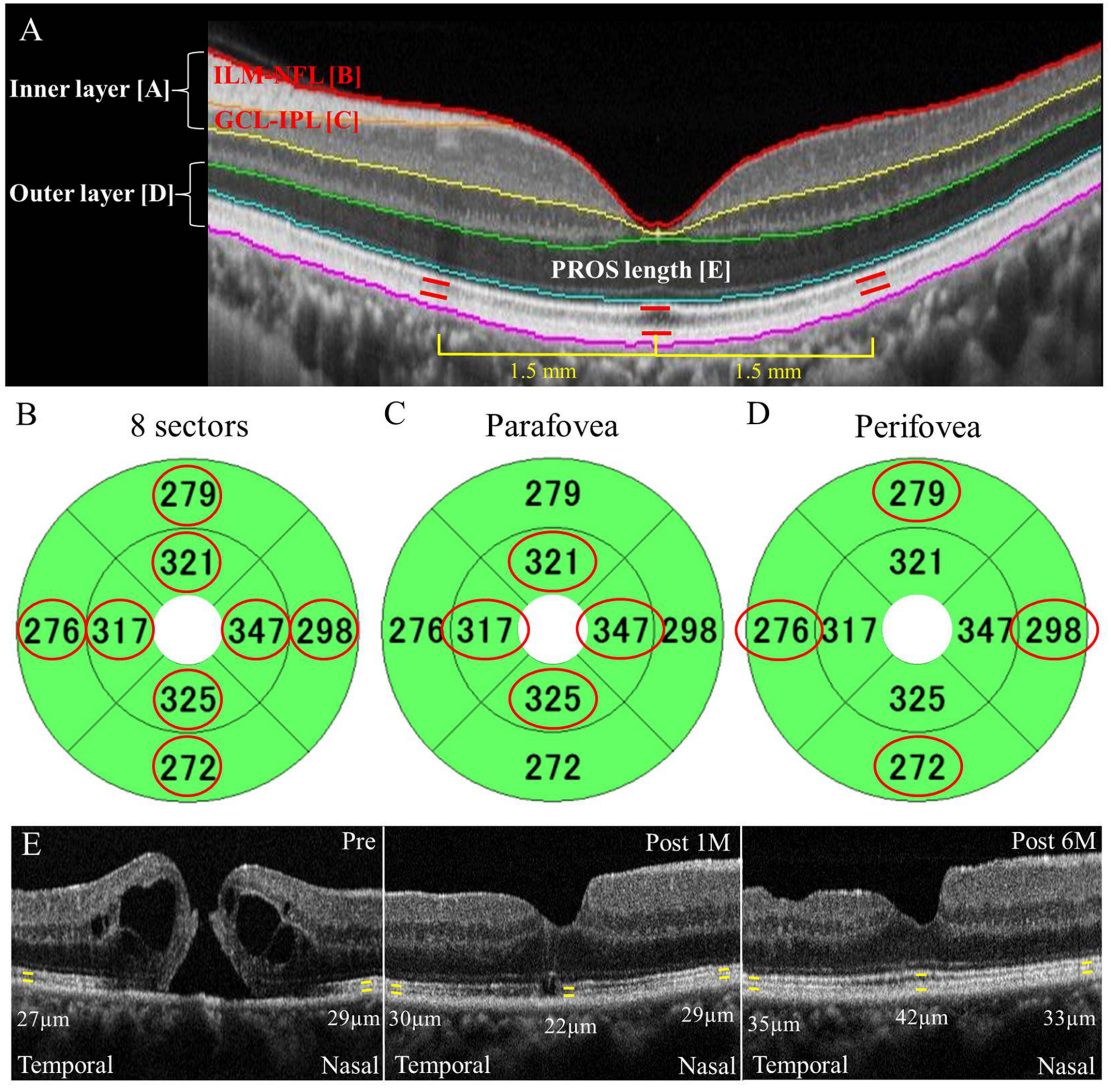
Measurements of inner and outer layer thicknesses using optical coherence tomography C-scan in eyes with an indiopathic macular hole. (A) Layer-by-layer measurements of the retina in a healthy eye. Thicknesses in the whole (ILM-RPE), inner (ILM-IPL [A], ILM-NFL [B], and GCL-IPL [C]), and outer (ONL-RPE [D]) layers measured on the basis of each colored line showing the border of automatically segmented layers. The PROS length [E] at the fovea and at a distance of 1.5 mm away from the fovea manually evaluated using B-scan. ILM: internal limiting membrane, RPE: retinal pigment epithelium, IPL: inner plexiform layer, NFL: nerve fiber layer, GCL: ganglion cell layer, ONL: outer nuclear layer, PROS: photoreceptor outer segment. (B-D) Overall retinal thicknesses (B) and the thicknesses of the inner 4 sectors (C) and outer 4 sectors (D) defined as the mean of the map sector values circled in red. (E) PROS length manually measured using vertical and horizontal scans through the central fovea at 5 spots: the fovea and the parafoveal sites (superior, inferior, nasal, and temporal) at a distance of 1.5 mm from the fovea. Representative case (Case 3) showing PROS length at the fovea and the temporal and nasal spots, pre-operatively and at 1 and 6 months post-surgery.

The *en face* C-scans of the macula (thickness map with the diameter of 6.0 mm) were obtained with OCT. Macular map images were automatically evaluated by the OCT software that provided thickness data for the whole retina (ILM to retinal pigment epithelium [RPE]), the inner retina (ILM to IPL; Fig 1A [A]), and the outer retina (outer nuclear layer [ONL] to RPE; Fig 1A [D]). Layer-by-layer measurements were made on each of the nine OCT map sectors, and eight sectors except the sector at the center were used for mean calculations for each of whole, inner, and outer retinal thicknesses, because the pre-operative presence of MH prevented thickness calculations for the centered one sector (within 1 mm in circle diameter; Fig 1B). Detailed analyses of inner retinal thickness were also performed at each time point including ILM-NFL thickness (Fig 1A [B]), ganglion cell layer (GCL)-IPL thickness (Fig 1A [C]), and inner layer (ILM-IPL) thickness in the inner 4 sectors of the parafoveal area (within 1–3 mm in annular diameter; Fig 1C, circled in red) and in the outer 4 sectors of the perifoveal area (within 3–6 mm in annular diameter; Fig 1D, circled in red). Mean thickness values of all the parameters measured were compared before surgery and 1, 3, and 6 months post-operatively.

Morphological changes, specifically at the fovea, were investigated using conventional cross-sectional B-scan images that allowed visualization of the ELM, the EZ, and the IZ. The photoreceptor outer segment (PROS) length at the fovea was measured manually using the scanner’s in-built caliper tool at each phase except pre-operatively (Fig 1A [E] and 1E), and the correlation between BCVA and PROS length at 6 months was determined. In addition, the parafoveal PROS length values at the superior, inferior, nasal, and temporal sites 1.5 mm apart from the center of the fovea were also measured at each time point. We evaluated the PROS length as 0 μm when the EZ at the fovea or parafovea was invisible post-operatively. Two of the authors (Y.H. and K.H.) performed an independent blind evaluation of the OCT images, without any knowledge of the subject’s clinical information. The repeatability of PROS length measurements was assessed by one author (Y.H.), who performed two independent measurements of PROS length from 6-month OCT images in all eyes at interval of one month.

### Statistical Analyses

All results are expressed as mean ± standard deviation. All results of the BCVA were converted to the logarithm of the minimal angle resolution (logMAR) for analyses. Friedman’s test and Scheffe’s paired comparison test were used for examining the sequential changes in BCVA and in the retinal layer thickness. Spearman’s rank test was used to determine the correlation between BCVA and PROS length changes. Statistical significance was defined as *P* value < 0.05.

## Results

### Post-operative Changes in Visual Acuity

**Fig 2:**
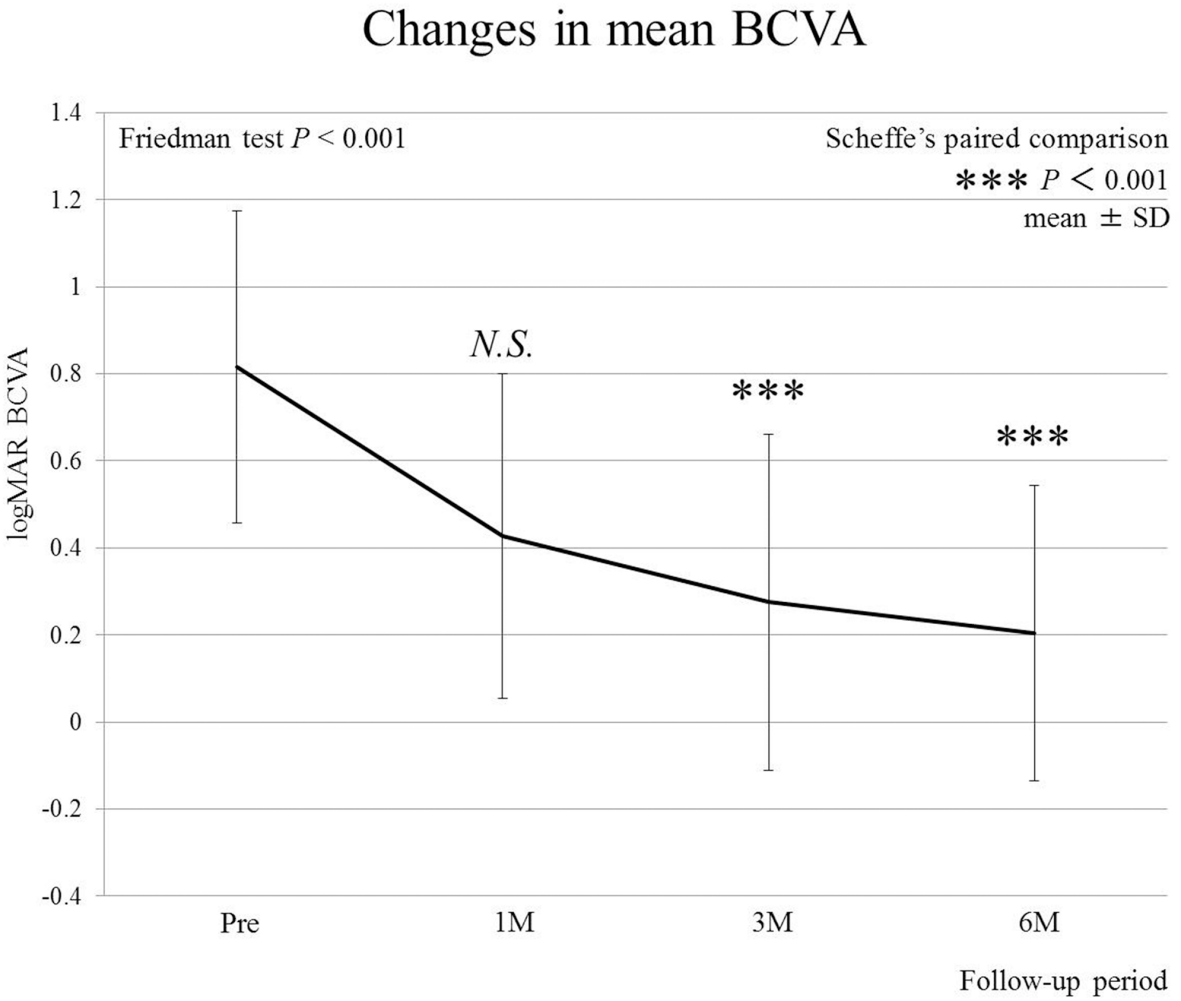
Sequential changes in best-corrected visual acuity (BCVA) after surgery. Significant improvements in BCVA obtained at 3 and 6 months following surgery (*P*<0.001, respectively).

None of the patients in this study experienced vision-threatening surgical or post-operative complications. Mean logMAR visual acuity was 0.81 ± 0.35, 0.43 ± 0.36, 0.27 ± 0.38, and 0.19 ± 0.34 before surgery and at 1, 3, and 6 months after surgery, respectively (Fig 2). This improvement was statistically significant at 3 and 6 months following surgery.

### Post-operative Changes in Retinal Thickness

Post-operative changes in retinal layer thicknesses are summarized in Table 2. Whole retinal thickness except for the fovea progressively decreased from the pre-operative value of 327.8 ± 25.8 μm to 312.1 ± 16.3 μm at 6 months after surgery, showing a statistically significant difference at the final evaluation.

**Table 2:** Post-operative Changes in Retinal Layer Thickness.

	Pre	Post			Friedman test	Scheffe's paired comparison (*P* value)		
		1M	3M	6M	(*P* value)	1M	3M	6M
Whole layer	327.8 ± 25.8	322.1 ± 11.8	316.7 ± 11.5	312.1 ± 16.3***	<0.001	0.99	0.46	0.0001
Inner layer								
8 sectors	108.6 ± 13.2	102.4 ± 8.0	98.4 ± 8.4**	98.7 ± 9.5*	<0.001	0.92	0.001	0.02
ILM-NFL	32.8 ± 7.4	33.5 ± 4.2	31.2 ± 4.7	31.5 ± 5.4	0.12	0.21	0.99	0.98
GCL-IPL	75.7 ± 7.7	68.3 ± 8.0**	67.1 ± 8.6***	67.2 ± 8.9**	<0.001	0.006	<0.001	0.002
Parafovea (4 sectors)	119.3 ± 19.8	107.4 ± 9.0	102.7 ± 9.7***	103.7 ± 10.6**	<0.001	0.25	<0.001	0.01
Perifovea (4 sectors)	98.1 ± 9.7	97.4 ± 9.1	94.1 ± 9.8	93.7 ± 11.1	0.003	0.92	0.15	0.15
Outer layer	128.8 ± 6.9	137.6 ± 6.4***	138.2 ± 6.4***	136.7 ± 6.0**	<0.001	<0.001	<0.001	0.002
PROS length								
Fovea	ND	17.3 ± 16.2	31.8 ± 11.2*	36.6 ± 10.2***	<0.001	ND	0.01	<0.001
Nasal	26.5 ± 3.9	29.6 ± 4.4	33.9 ± 4.3***	33.4 ± 2.9***	<0.001	0.27	<0.001	<0.001
Temporal	27.6 ± 3.9	30.1 ± 5.0	32.3 ± 4.9***	32.6 ± 3.8***	<0.001	0.18	<0.001	<0.001
Superior	26.1 ± 4.6	30.5 ± 4.6	34.0 ± 4.0***	33.5 ± 3.8***	<0.001	0.11	<0.001	<0.001
Inferior	23.6 ± 4.7	28.6 ± 4.8*	31.2 ± 4.0***	32.9 ± 2.7***	<0.001	0.02	<0.001	<0.001

ILM = internal limiting membrane; NFL = nerve fiber layer; GCL = ganglion cell layer; IPL = inner plexiform layer; PROS = photoreceptor outer segment; ND = not done;

*P<0.05,

**P<0.01,

***P<0.001.

**Fig 3:**
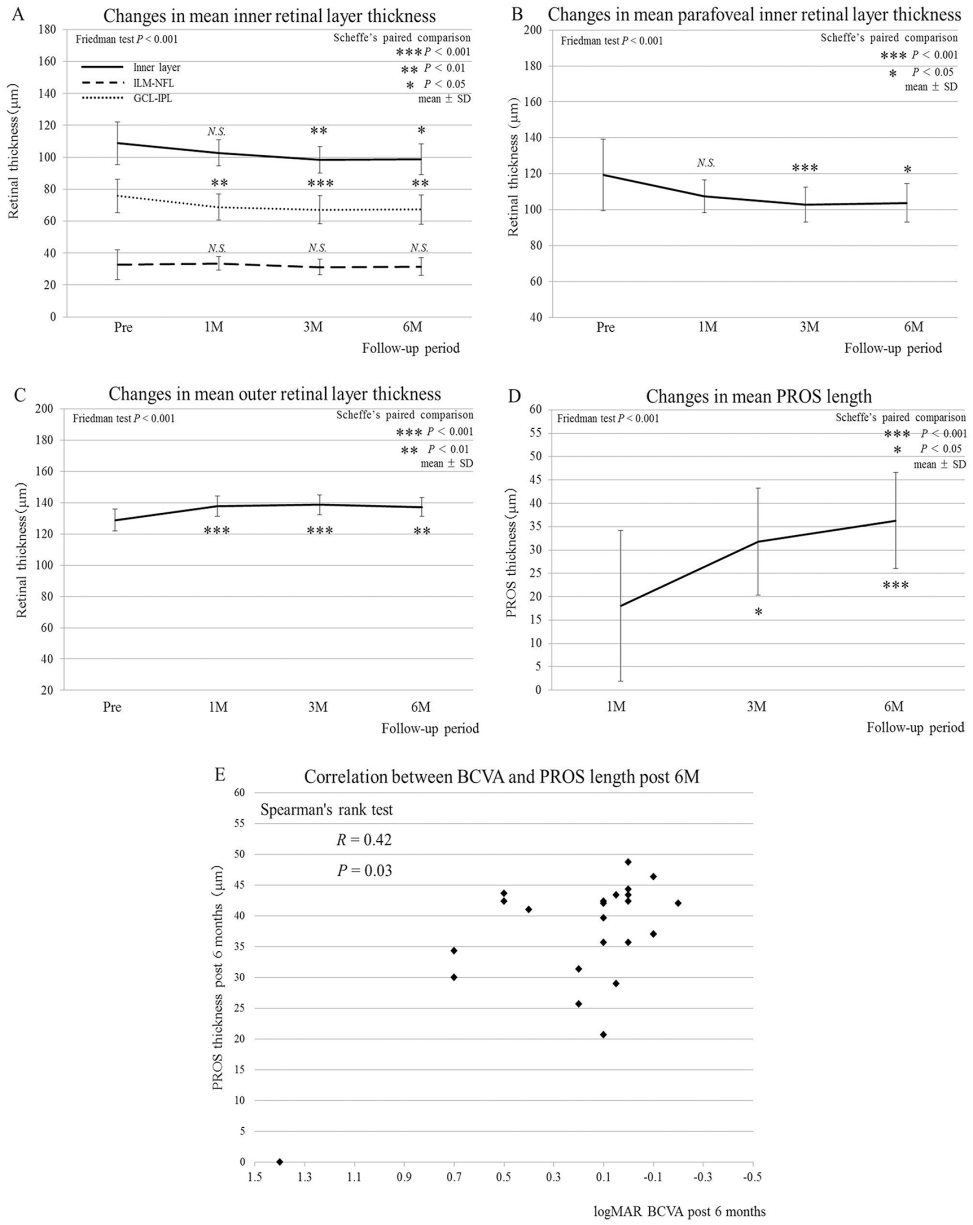
Post-operative changes in mean thickness in individual retinal layers and PROS length together with its correlation with BCVA. (A) The inner retinal layer (ILM-IPL, 8 sectors) showing a significant decrease in thickness at 3 and 6 months after surgery (*P* = 0.001, *P* = 0.02, respectively); the ILM-NFL layer (8 sectors) with no significant change at any of the time points measured; the GCL-IPL-layer (8 sectors) showing a significant reduction in thickness at 1 month and thereafter (*P* = 0.006, *P*<0.001, *P* = 0.002, respectively). (B) The inner layer (ILM-IPL, 4 sectors) at the parafovea showing a significant decrease in thickness at 3 and 6 months after surgery (*P*<0.001, *P* = 0.01, respectively). (C) The outer layer (8 sectors) showing a significant increase in thickness at 1 month and later (*P*<0.001, *P*<0.001, *P* = 0.002, respectively). (D) The mean foveal PROS length showing a significant increase at 3 and 6 months compared with the 1-month value (*P* = 0.01, *P*<0.001, respectively). (E) A positive correlation found between BCVA and the foveal PROS length at 6 months after surgery (*R* = 0.42, *P* = 0.03).

The thickness of the inner layer also progressively decreased from 108.6 ± 13.2 μm to 98.7 ± 9.5 μm at 6 months after surgery. This decrease became statistically significant at 3 and 6 months following surgery (Fig 3A). Interestingly, the thickness of the ILM-NFL layer did not change throughout the course of the study (Fig 3A), whereas the thickness of the GCL-IPL layer significantly decreased at all the time points, measuring 75.7 ± 7.7 μm pre-operatively and 67.2 ± 8.9 μm at 6 months after surgery (Fig 3A), showing that the sequential thinning of the inner layer after surgery depended mainly on the GCL-IPL layer but not the ILM-NFL layer.

The thickness of the parafoveal inner layer (Fig 1C) significantly decreased at 3 and 6 months after surgery (Fig 3B). In contrast, the thickness of the perifoveal inner layer (Fig 1D) did not change significantly at any of the time points measured (Table 2). Thus, the post-operative thinning of the inner retinal layer appeared to be confined within the parafoveal area.

Contrary to the overall inner layer changes, the thickness of the outer layer except for the fovea increased from 128.8 ± 6.9 to 136.7 ± 6.0 μm at 6 months. This increase was statistically significant at all time points measured (Fig 3C). The thickness of both parafoveal and perifoveal outer layers also significantly increased post-operatively (data not shown).

### Retinal Morphological Changes

A cystoid spaces at the inner nuclear layer (INL) to the outer plexiform layer (OPL) were observed pre-operatively in 23 of the 24 MH eyes, which resolved in all eyes following successful MH closure (Fig 1E). Interestingly, none of the affected eyes demonstrated cystoid spaces at the GCL-IPL layer, although this layer was the main contributor to the post-operative inner layer thinning (Fig 3A).

Post-operative outer morphological changes in the fovea are summarized in Table 1. One month after surgery, the ELM was normal in 5 eyes (20.8%), discontinuous in 17 eyes (70.8%), and invisible in 2 eyes (8.4%). The EZ was normal in 0 eyes, discontinuous in 15 eyes (62.5%), and invisible in 9 eyes (37.5%). The IZ was normal in 0 eyes, discontinuous in 4 eyes (16.6%), and invisible in 20 eyes (83.4%). At 6 months after surgery, the ELM was normal in 13 eyes (54.1%), discontinuous in 10 eyes (41.6%), and invisible in 1 eye (4.3%). The EZ was normal in 10 eyes (41.6%), discontinuous in 13 eyes (54.1%), and invisible in 1 eye (4.3%). The IZ was normal in 3 eyes (12.5%), discontinuous in 12 eyes (50.0%), and invisible in 9 eyes (37.5%). These observations on the outer retina indicated a substantial structural improvement after MH surgery.

### Correlation Between PROS Length and Visual Acuity

Post-operative changes in PROS length in MH eyes are summarized in Table 2. In agreement with the outer retinal layer changes (Fig 3C), PROS length at the fovea significantly increased 3 and 6 months after surgery when compared to 1 month post-surgery (Fig 3D). Additionally, PROS length values at all parafoveal sites measured (i.e., nasal, temporal, superior, and inferior) also gradually increased post-operatively, all of which showed statistical significance at 3 and 6 months when compared to the baseline values (Table 2).

The correlation coefficient for PROS length measurement repeatability was high (*R* = 0.86, *P*<0.001), indicating a good agreement between the two individual manual measurements. The 3-sigma repeatability of the image analyses for PROS length measurements was reported to be 2.8 μm [12].

BCVA and foveal PROS length were positively correlated at 6 months after surgery (Fig 3E; *R* = 0.42, *P* = 0.03). Importantly, no significant correlation was detected between BCVA and any of the other thickness parameters obtained post-operatively that included whole, inner (8 sectors, parafoveal and perifoveal regions, and the GCL-IPL and ILM-NFL layers), and outer retinal layers, in addition to the parafoveal PROS length values at all the 4 sites measured.

## Discussion

Recent advances in ocular imaging technology have successfully enabled more and more detailed analyses to evaluate morphological and functional prognosis in MH formation [13] and repair [14,15]. To the best of our knowledge, the present study is the first to reveal sequential changes in thickness of inner and outer retinal layers in the macular area following PPV for idiopathic MH and a correlation between photoreceptor anatomical recovery and visual outcomes. Following surgery, the whole of the retina except the fovea thinned, with most of the thinning associated with the inner retinal layer, especially the GCL-IPL layer in parafoveal area. In contrast, the outer retinal layer thickened mainly from substantial thickening of the PROS, which lengthened over time in not only the fovea but also the parafoveal area. Importantly, the post-operative BCVA was correlated exclusively with the 6-month post-surgery PROS length at the fovea, but not with any of other thickness data concurrently collected.

In the present study, we found that the layer responsible for the decrease in inner retinal thickness following successful MH closure was confined to the GCL to IPL region in the parafoveal area. However, the intra-retinal cystoid spaces pre-operatively seen at the edge of MH were reported to localize histopathologically in the INL and the OPL [16], both of which constitute the middle retinal layers. Indeed, we did not observe any apparent cystoid spaces in the GCL-IPL region in any eyes pre-operatively. Therefore, these observations suggest that the GCL-IPL layer was subclinically involved in MH eyes. As a possible mechanism, the effect of BBG on the specific region might be considerable; however, studies have reported that BBG exercises no toxic actions to retinal ganglion cells both *in vivo* and *in vitro* [17,18]. Results have shown that BBG can inhibit the apoptosis of photoreceptor cells and may be a good candidate drug for neuroprotective therapy [19]. Therefore, retinal cytotoxicity associated with BBG use is theoretically rare.

Surprisingly, in contrast to the GCL-IPL layer thinning after surgery, the most superficial (ILM-NFL) layer thickness did not show any significant changes during follow-up in our cases applied with BBG. This is supported by and consistent with recently published data showing that BBG-assisted MH surgeries eventually maintained the baseline level of NFL thickness [20]. More importantly, the degree of GCL-IPL thinning currently observed, approximately 10 μm, is equivalent to that of the NFL-IPL thinning after MH surgery assisted by BBG [21]. These recent analyses on inner retinal layers [20, 21] further validate our measurements. Thus, retinal mechanical and cytotoxic damage as a consequence of ILM removal is likely to be minimal or negligible.

In human retina, retinal capillary networks surrounding the fovea are present in the following 4 layers: the NFL, the GCL-superficial portion of the IPL, the deep portion of the IPL to the superficial portion of the INL, and the deep portion of the INL [22]. Accordingly, cystoid spaces commonly observed in the middle (INL-OPL) layer in MH eyes are thought to stem from the mechanical breakdown of the deeper retinal capillary networks during MH formation, i.e., the process of dehiscence at the central fovea lacking the inner retinal layers. On the other hand, the current data revealed the subclinical but significant changes in thickness of the inner layer without any apparent cysts pre-operatively. In a recent study using the OCT C-scan, we have demonstrated that the inner retinal layer subclinically thickened in the acute stage of multiple evanescent white dot syndrome, a known outer layer disorder, possibly due to associated retinal vasculitis [23]. Similarly, the currently observed inner (GCL-IPL) layer thinning after surgery appeared to be derived from the pre-operative subclinical breakdown of the more superficial capillary networks due to an unspecified etiology associated with MH formation.

The current study has also demonstrated a significant increase in the macular outer layer thickness following surgery, which was attributable to an increase in PROS length positively correlated with the post-operative visual acuity. Actually, an association between PROS length and visual function in macular diseases has been reported. Post-operative BCVA showed a correlation with PROS length but not with central retinal thickness in eyes with diabetic macular edema [24]. PROS length has also been shown to be significantly correlated with BCVA after PPV in patients with idiopathic ERM [12,25]. We have recently reported that the inner retina thinned but the outer retinal layer thickened after ERM surgery and that the PROS elongation was positively correlated with visual recovery [25]. In accordance with the ERM data [12,25], the present results demonstrated that post-operative visual acuity in MH eyes depends exclusively on the recovery of disrupted foveal photoreceptors.

In the present study, the PROS length increase was found in both the foveal and the parafoveal regions. These observations suggest that the outer retinal layers in eyes with MH were impaired across a wider area of the retina than the fovea per se. Previously, decreased responses in multifocal electroretinography were detected not only at the fovea but also in the wider area within 1.6 disc diameters in MH eyes [26]. These results suggest that the outer retinal layers of the surrounding regions of the fovea are also functionally impaired in pre-surgery MH eyes. Our data provide significant morphological evidence that the outer retinal layers surrounding the fovea are also affected in MH eyes.

The primary limitations of this study include its retrospective design and the relatively small sample size. From lack of a control group, it would be difficult to definitely determine factors contributing to the post-operative thinning of inner layers. In the present case series, four retinal specialists performed PPV. We could not completely exclude the possibility that the difference in the surgeons’ technique of ILM peeling or related surgical trauma, even if subtle, may have affected post-operative changes in inner retinal layers, although no significant difference in the NFL thickness detected between pre- and post-operatively. Lastly, the resolution of the current OCT technology precludes the accurate measurement of ILM thickness alone.

## Conclusions

Our current investigation newly demonstrated detailed layer-by-layer results on sequential changes in retinal thickness following PPV for MH, together with their implications for visual function. Post-operatively, retinal inner layers other than the nerve fiber layer thinned, suggesting the pre-operative subclinical thickening of the inner layers even without any apparent cysts before surgery. In contrast, retinal outer layer thickness significantly increased after surgery, potentially as a result of foveal PROS elongation linking exclusively to visual improvement in MH eyes. Future studies are required to further elucidate the relationship between structure and function to better understand the pathogenesis of idiopathic MH.
